# Does MRI Have a Role in the Preoperative Staging of Penile Cancer?

**DOI:** 10.7759/cureus.56016

**Published:** 2024-03-12

**Authors:** Muhammad Iqbal, Mostafa Shendy, Anna McClune, Wail Mohamed, Anthony Shanahan, Balan Palaniappan, Gareth Brown

**Affiliations:** 1 Urology, Cwm Taf Morgannwg University Health Board, Royal Glamorgan Hospital, Llantrisant, GBR; 2 Urology, Mid Cheshire Hospitals NHS Foundation Trust, Leighton Hospital, Crewe, GBR; 3 Radiology, Cwm Taf Morgannwg University Health Board, Royal Glamorgan Hospital, Llantrisant, GBR

**Keywords:** mri, squamous cell carcinoma, tumour staging, systematic review, staging, penis, corpora cavernosa, carcinoma, mri penis, penile cancer

## Abstract

Background

Penile cancer is a rare malignancy usually requiring surgery to achieve oncological control of the primary tumour but often at the expense of functional length. The presenting stage of the primary is a crucial factor in determining the most appropriate surgical procedure. Accurate preoperative staging is essential, and current modalities include clinical and radiological assessment. Clinical staging can, however, be hampered by patient body habitus and unreliable for more advanced T4 tumours, whereas radiological staging allows for more detailed identification of tissue planes and tumour involvement. There is no clear consensus on the preferred imaging technique, although, in the current European Association of Urology penile cancer guidelines, MRI is recommended with the use of ultrasound when MRI is not available. It was recommended that having the penis in an erect state by the administration of intra-cavernosal prostaglandin gave a more detailed picture enabling a greater predictor of corporal involvement. Recent studies have, however, suggested that there may be no such advantage.

Methodology

A retrospective review was conducted of all patients who underwent surgery for penile cancer comparing the preoperative MRI stage with the final pathological stage between July 2009 and June 2023. In addition to the MRI, patients were given an intra-cavernosal injection of prostaglandin E1 to induce tumescence unless otherwise indicated. All imaging was reported by a single consultant uro-radiologist with surgery undertaken by a single surgeon and pathology reviewed through the supra-regional penile multidisciplinary team.

Results

A total of 136 penile cancer patients were included in the review. Within this cohort, 98 patients had an MRI without intra-cavernosal prostaglandin and the number who had Ta, T1, T2, T3 and T4 histopathological stages was 3, 31, 45, 18, and 1, respectively. The preoperative MRI stage had a low agreement with the final histological stage for early tumours, with sensitivities and specificity of 35% and 97% for T1 and 56% and 80% for T2, respectively. Sensitivity and specificity increased for cavernosal involvement at 83% and 95%, respectively. In addition, a further 38 patients had an MRI in conjunction with an injection of prostaglandin E1 which failed to show any diagnostic improvement in sensitivity or specificity in the preoperative MRI stage.

Conclusions

The use of MRI as a preoperative modality for staging penile cancer performs best for identifying tumour involvement of the cavernosal bodies. Performing the MRI with the penis erect with the use of an intra-cavernosal injection did not offer any additional benefit in accurately staging penile cancer.

## Introduction

Penile cancer is a rare malignancy with a rising incidence. It is commonly seen in men between the ages of 50 and 70 years. Global variation is noted, with the reported rates of penile cancer in developed countries being lower with around 0.94 per 100,000 males in Europe and 0.5 per 100,000 in the United States and much higher in developing countries such as South America, Southeast Asia, and parts of Africa where penile cancer accounts for 10% of male malignancies [1]. Risk factors for penile cancer include human papillomavirus infection, phimosis, smoking, poor hygiene, and chronic inflammatory conditions.

Squamous cell carcinoma is the predominant neoplastic type responsible for 95% of cases, with the majority arising from the glans (48%), followed by the prepuce (21%), both glans and prepuce (9%), the coronal sulcus (6%), and less than 2% of cases isolated to the shaft. Surgery is the mainstay of treatment with the aim, whenever possible, of achieving a balance between penile-preserving surgery versus successful oncological control [2].

Penile amputation is associated with significant functional, sexual, and psychological side effects, despite providing good oncological control. Organ preservation techniques have, therefore, become popular with reported superior functional outcomes and improved quality of life. Nevertheless, there is potentially an increased risk of local recurrence after organ-sparing surgery in comparison with penile amputation. The type of preferred surgical treatment depends on factors such as tumour grade and stage, patient preference, and anatomical considerations. Accurate primary tumour staging is essential for determining the most appropriate organ-preserving surgery [3].

Methods of staging the primary tumour include clinical examination and imaging modalities such as MRI. The 2017 revision of penile cancer staging by the American Joint Committee on Cancer-Tumour Node and Metastasis (AJCC-TNM) eighth edition reclassified corpora cavernosa invasion from the previous T2 to T3 stage, recognising the significance of corporal involvement as a risk factor for micro-metastatic inguinal lymph node involvement in clinically impalpable nodes [4]. Therefore, not only is accurate preoperative staging relevant in planning treatment but also in future planning of inguinal lymph node management. MRI offers increased soft tissue contrast and multiplanar capabilities, which generates optimal images of all penile compartments making it an ideal modality for penile cancer staging. The European Association of Urology (EAU) suggests that for low-grade tumours (T1 and T2), MRI is on par with clinical staging, but that MRI may be more useful in determining the invasion of cavernous bodies and detecting T3 disease [5]. MRI can exclude invasion of the corpora cavernosa, especially if penis preservation is planned. Some studies suggest the concurrent administration of intra-cavernosal prostaglandin E1 increases sensitivity in MRI staging; however, a recent study by Ghosh et al. (2021) [6] and a systematic review and meta-analysis by Krishna et al. (2022) reported no such advantage [7].

Upon reviewing the literature, there are only a small number of published studies reporting the use of MRI in staging penile cancer all of which are limited by small patient numbers. This study aimed to review whether MRI still played a role in the preoperative staging of penile cancer and would, therefore, be a useful tool in planning penile surgery. A secondary aim was to examine whether the use of intra-cavernosal prostaglandin improved staging accuracy. The preoperative MRI stage was compared with the final histopathology stage.

## Materials and methods

Study design

A retrospective review was conducted of all patients who underwent a preoperative staging MRI of their penile cancer in a single centre between July 2009 and June 2023. MRI results were compared with final histopathological staging following definitive treatment of the primary malignancy. All patients who underwent a staging MRI for penile cancer were included. Patients were excluded if they had a diagnosis of penile intraepithelial neoplasia or a penile preputial carcinoma, were treated with radiotherapy and therefore no final pathological staging was available, or were not a candidate for an MRI due to claustrophobia or the presence of a cardiac pacemaker or implantable cardiac defibrillator.

Ethical approval and informed consent

Ethical approval was granted by the Audit Department of the Royal Glamorgan Hospital (approval number: Urology/CA/2023-24/02). Given the retrospective nature of the study and the non-utilization of patients’ personal data, written consent from the patients was deemed unnecessary. All patients were treated per the current guidance and best practices recommended by the EAU. All patients were fully informed during their treatment pathway. A diagnosis of cancer was confirmed in all cases by biopsy. The use of intraoperative frozen sections ensured that oncological clear margins were achieved when treating the primary [8]. All data collected on the MRI report with the corresponding final histopathology was anonymised, with patient-identifiable information removed before being reviewed and analysed by the researcher.

Study procedure

All patients underwent an MRI scan using a 1.5 T Siemens Aera/Sola machine with a body coil. All patients received an intra-cavernosal prostaglandin E1 (Alprostadil) injection before scanning unless considered unsuitable, e.g., patients with a fungating mass, buried penis, or those with contraindications to the injection. The penis was then placed on the abdominal wall and scanned in high resolution (3 mm slice thickness) T2 turbo spin echo (TSE) scan field of view in three orthogonal planes. T1 TSE transverse and coronal planes included the whole pelvis and inguinal regions to assess the primary and local lymph nodes. All scans were reported by a single uro-radiologist with experience in reporting penile MRIs. Patients then proceeded to have definitive surgical treatment with all pathological specimens formally reviewed through the supra-regional penile cancer multidisciplinary team meeting. Tumours were staged according to the Union for International Cancer Control/AJCC eighth edition TMN classification (Table 1).

**Table 1:** Union for International Cancer Control/American Joint Committee on Cancer eighth edition Tumour Node and Metastasis classification.

T - Primary tumour		
TX	Primary tumour cannot be assessed	
T0	No evidence of a primary tumour	
Tis	Carcinoma in situ (penile intraepithelial neoplasia)	
Ta	Non-invasive verrucous carcinoma*	
T1	Tumour invades subepithelial connective tissue	
	T1a	Tumour invades subepithelial connective tissue without lymphovascular invasion or perineural invasion and is not poorly differentiated
	T1b	Tumour invades subepithelial connective tissue with lymphovascular invasion or perineural invasion or is poorly differentiated
T2	Tumour invades corpus spongiosum with or without invasion of the urethra	
T3	Tumour invades corpus cavernosum with or without invasion of the urethra	
T4	Tumour invades other adjacent structures	
N - Regional lymph nodes		
cNX	Regional lymph nodes cannot be assessed	
cN0	No palpable or visibly enlarged inguinal lymph nodes	
cN1	Palpable mobile unilateral inguinal lymph node	
cN2	Palpable mobile multiple or bilateral inguinal lymph nodes	
cN3	Fixed inguinal nodal mass or pelvic lymphadenopathy, unilateral or bilateral	
M - Distant metastasis		
cM0	No distant metastasis	
cM1	Distant metastasis	

Statistical analysis

The patients were divided into two groups depending on whether they were given an intra-cavernosal injection. The non-injection group included 98 patients who had only MRI staging of their penile cancer, and the injection group included 38 patients who underwent an MRI with the addition of an intra-cavernosal injection of prostaglandin E1. The validity of MRI in staging penile cancer was assessed by comparing radiological and histopathological staging and analysed by calculating sensitivity, specificity, and positive and negative predictive values for both groups.

## Results

The number of patients in the non-injection group with Ta, T1, T2, T3, and T4 histopathology stages was 3, 31, 45, 18, and 1, respectively. The sensitivity of MRI in correctly staging penile cancer was low for less invasive stages at 0% for Ta, 35% for T1, and 56% for T2. This reduced sensitivity was associated with an almost equal number of under-staging and over-staging for T1 and T2 disease. Of 31 patients with histopathological pT1 disease, the MRI correctly staged 11, with 9 cases failing to identify cancer (Tx) and 11 incorrectly over-staged (9 staged as T2 and 2 staged as T3). For confirmed pathological T2 stage penile cancer, the MRI accurately staged 25 out of 45 cases, with 11 under-staged (9 as Tx and 2 as T1) and a further 9 over-staged (8 as T3 and one as T4) (Tables 2, 3).

**Table 2:** Comparison of the preoperative MRI and histopathology stages for patients in the non-injectable group, who only underwent MRI.

				Histology		
MRI	Ta	T1	T2	T3	T4	Total
TX	3	9	9	1	0	22
T1	0	11	2	0	0	13
T2	0	9	25	2	0	36
T3	0	2	8	15	0	25
T4	0	0	1	0	1	2
Total	3	31	45	18	1	98

**Table 3:** Analysis demonstrating the MRI sensitivity, specificity, PPV, and NPV for the non-injection group. PPV: positive predictive value; NPV: negative predictive value

Pathological T stage	PPV (%)	NPV (%)	Sensitivity (%)	Specificity (%)
Ta	0	99	0	100
T1	85	77	35	97
T2	69	68	56	80
T3	83	88	83	96
T4	5	100	100	99

The number of patients in the injection group (who had an MRI following an intra-cavernosal prostaglandin E1 injection) with histologically confirmed T1 stage cancer was 21, with 9 having T2, 7 diagnosed with T3, and 1 with T4 penile cancer. For this group, similar results were recorded with MRI sensitivity low for T1 and T2 stage cancer at 5% and 44%, respectively. For patients with pT1 disease, MRI diagnosed only one case correctly, with 5 over-staged (3 as T2 and 2 as T3) and the remaining 15 cases not detected. For the 9 men with pT2 disease, MRI reported correctly 4 cases with 4 under-staged (2 staged as Tx and 2 as T1) and one case over-staged as T3.

MRI sensitivity was greater for T3 and T4 tumours at 86% and 100%, respectively, with the specificity for all stages being high (Tables 4, 5).

**Table 4:** Comparison of the preoperative MRI and histopathology stages for patients in the injectable group, who underwent MRI with an intra-cavernosal prostaglandin injection.

			Histology			
MRI	Ta	T1	T2	T3	T4	Total
TX	0	15	2	1	0	18
T1	0	1	2	0	0	3
T2	0	3	4	0	0	7
T3	0	2	1	6	0	9
T4	0	0	0	0	1	1
Total	0	21	9	7	1	38

**Table 5:** Analysis demonstrating MRI sensitivity, specificity, PPV, and NPV for the injection group. PPV: positive predictive value; NPV: negative predictive value

Pathological T stage	PPV (%)	NPV (%)	Sensitivity (%)	Specificity (%)
Ta	0	100	0	100
T1	33	41	5	86
T2	57	83	44	89
T3	67	97	86	90
T4	100	100	100	100

## Discussion

Penile cancer, although a relatively rare malignancy, carries significant morbidity and mortality. Surgery is the mainstay of treatment to achieve complete cancer removal while trying to optimise organ preservation whenever possible. Physical assessment in penile cancer can be challenging and affected by factors such as concurrent infections or variations in body habitus. Accurate preoperative staging is imperative in planning penile cancer treatments. In recent years, MRI has evolved to become an important adjunct to the physical examination or, in some equivocal cases, as an alternative investigation. In addition, it is important to recognise T2+ disease which carries an increased risk for micro-metastatic inguinal node involvement in patients with impalpable nodes who would then be considered for prophylactic groin node dissection or sentinel lymph node biopsy [9]. The use of an intra-cavernosal injection of prostaglandin E1 to attain an erection during MRI was thought to further improve the staging accuracy of the primary. The resultant distended T2-hyperintense corpora cavernosa with a high resolution of contrast allows for better visualisation of the tissue planes [10]. However, intra-cavernosal prostaglandin E1 injections may cause pain at the injection site, bruising, haematoma, and priapism [11]. As a consequence, prostaglandin E1 injections are not universally utilised.

Our results show that MRI sensitivity and specificity in correctly staging penile cancer increased as the T stage progressed, particularly in diagnosing tumours with cavernosal involvement. Although the sensitivity of MRI is low in correctly staging penile cancer, its performance in predicting the stage accurately is higher, as evidenced by the higher positive predictive values (Table 3).

Our observations align with earlier studies in the literature, including investigations by Scardino et al. and Petralia et al., which also emphasised the significance of MRI in staging the primary tumour (T-staging) and its potential for planning organ preservation. Both of these studies reported commendable accuracy rates, reaching 88.8% and 92.3%, respectively. Nevertheless, it is crucial to recognise that in these studies, MRI was conducted following pharmacologically induced penile erection and involved a limited patient cohort (9 and 13 patients, respectively) [12,13]. More recently, another study by Hanchanale et al. evaluated the accuracy of MRI in predicting loco-regional invasion in penile cancer. This study demonstrated sensitivity and specificity for predicting corpus cavernous invasion at 62.5% and 86.9%, respectively, in a series of 100 patients [14].

Recent advancements have led to further exploration of the capabilities of MRI in penile cancer. For instance, a study by Ghosh et al. aimed to assess ‘non-erect’ MRI in staging and preoperative evaluation of penile carcinomas comparing the results to postoperative histopathology in a cohort of 50 patients. Their findings indicated high specificity and sensitivity for detecting the involvement of the corpus spongiosum, corpora cavernosa, and urethra, with values ranging from 87.5% to 100%. Moreover, MRI exhibited high sensitivity (89.6%) and specificity (100%) in predicting adequate disease-free penile length [6].

In a 2023 study by Switlyk et al., which involved 25 patients undergoing preoperative penile MRI without artificial erection, a noteworthy concordance was observed between MRI and histopathology, particularly in assessing the involvement of the corpus spongiosum (p = 0.002). An agreement was also noted for the involvement of the penile urethra and the tunica albuginea/corpus cavernosum (p < 0.001 and p = 0.007, respectively). While the agreement was less robust, it remained substantial for both overall T staging and N staging (p < 0.001 and p = 0.002, respectively) [15]. On the contrary Petraila et al. suggested that an MRI of the penis with an intra-cavernosal prostaglandin injection performed better than a clinical examination. This study included 13 patients who underwent an MRI of the penis with intra-cavernosal prostaglandin E1 injection. This study was limited to a small cohort and did not compare an MRI of the penis with and without intra-cavernosal prostaglandin E1 injections [15].

A recent systemic review by Krishna et al. included eight studies and 481 patients The sensitivity and specificity of MRI were 86% (95% confidence interval (CI) = 73-94%) and 89% (95% CI = 77-95%), respectively, in distinguishing ≤T1 from ≥T2 disease. The study reported that an MRI of the penis had a sensitivity and specificity of 80% (95% CI = 70-87%) and 96% (95% CI = 85-99%), respectively, in accurately diagnosing T3 disease. The sensitivity and specificity for MRI with versus without intra-cavernosal prostaglandin E1 Injection was 85% (95% CI = 71-92%) and 93% (95% CI = 77-98%) versus 86% (95% CI = 68-95%) and 84% (95% CI = 70-93%), respectively (p = 0.50). This review reported similar accuracy in local staging for MRI with and without artificial erection [7]. Similar findings were also noted in our study with no increase in sensitivity and specificity in patients who had an intra-cavernosal injection of prostaglandin E1 versus those who did not (Table 5). The current evidence from the aforementioned studies adds to our conclusion regarding the diagnostic accuracy of MRI in penile cancer, emphasising its growing importance in the clinical assessment of penile malignancy.

Although our review is limited by its retrospective design and relatively low sample size, the patient numbers are higher than most previously published studies. The high specificity shown in our results is not unexpected as MRI was used to stage an already confirmed cancer with clinical findings available to the reviewing radiologist. Our study suggests that MRI on its own is not a reliable diagnostic tool in staging early T1 and T2 penile cancers but has high accuracy in staging T3 tumours. A further prospective study comparing the clinical stage with radiology and histopathology may provide a clearer picture regarding the most appropriate staging modality.

## Conclusions

As a diagnostic test, MRI has a limited role in the preoperative staging of primary penile cancer, with many cancers under or over-staged, particularly for those with pathological T2 tumours or less. MRI demonstrated a higher sensitivity and specificity in accurately diagnosing cavernosal involvement and beyond. The administration of intra-cavernosal prostaglandin E1 injection provided no additional benefit in improving MRI radiological staging.
